# Thymidylate synthase drives the phenotypes of epithelial-to-mesenchymal transition in non-small cell lung cancer

**DOI:** 10.1038/s41416-020-01095-x

**Published:** 2020-10-07

**Authors:** Mohammad Aarif Siddiqui, Paradesi Naidu Gollavilli, Vignesh Ramesh, Beatrice Parma, Annemarie Schwab, Maria Eleni Vazakidou, Ramakrishnan Natesan, Ozge Saatci, Ida Rapa, Paolo Bironzo, Harald Schuhwerk, Irfan Ahmed Asangani, Ozgur Sahin, Marco Volante, Paolo Ceppi

**Affiliations:** 1 Department of Biochemistry and Molecular Biology, University of Southern Denmark, Odense, Denmark; 2 Interdisciplinary Center for Clinical Research (IZKF), Friedrich-Alexander University of Erlangen-Nuremberg, Erlangen, Germany; 3 Perelman School of Medicine, University of Pennsylvania, Philadelphia, PA USA; 4 Department of Drug Discovery and Biomedical Sciences, University of South Carolina, Columbia, SC USA; 5 Department of Oncology at San Luigi Hospital, University of Turin, Orbassano, Turin, Italy; 6 Department of Experimental Medicine-I, Friedrich-Alexander University of Erlangen-Nuremberg, Erlangen, Germany

**Keywords:** Non-small-cell lung cancer, Tumour biomarkers

## Abstract

**Background:**

Epithelial-to-mesenchymal transition (EMT) enhances motility, stemness, chemoresistance and metastasis. Little is known about how various pathways coordinate to elicit EMT’s different functional aspects in non-small cell lung cancer (NSCLC). Thymidylate synthase (TS) has been previously correlated with EMT transcription factor ZEB1 in NSCLC and imparts resistance against anti-folate chemotherapy. In this study, we establish a functional correlation between TS, EMT, chemotherapy and metastasis and propose a network for TS mediated EMT.

**Methods:**

Published datasets were analysed to evaluate the significance of TS in NSCLC fitness and prognosis. Promoter reporter assay was used to sort NSCLC cell lines in TS^HIGH^ and TS^LOW^. Metastasis was assayed in a syngeneic mouse model.

**Results:**

TS levels were prognostic and predicted chemotherapy response. Cell lines with higher TS promoter activity were more mesenchymal-like. RNA-seq identified EMT as one of the most differentially regulated pathways in connection to TS expression. EMT transcription factors HOXC6 and HMGA2 were identified as upstream regulator of TS, and AXL, SPARC and FOSL1 as downstream effectors. TS knock-down reduced the metastatic colonisation in vivo.

**Conclusion:**

These results establish TS as a theranostic NSCLC marker integrating survival, chemo-resistance and EMT, and identifies a regulatory network that could be targeted in EMT-driven NSCLC.

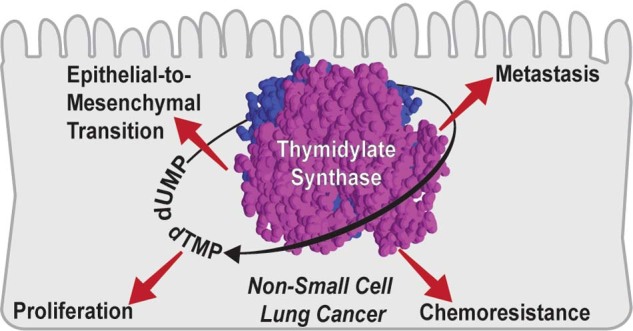

## Background

Epithelial-to-mesenchymal transition (EMT) is an embryonic process hijacked by epithelial-like carcinoma cells to gain mesenchymal-like phenotype. Oncogenic EMT is a gamut of functional changes, such as enhanced motility, invasiveness, stemness, aggressiveness and chemoresistance, and is a key determinant of metastasis. EMT is a complex cascade of molecular events engendered by master EMT transcription factors (EMT-TFs, ZEB1/2, SNAI1/2 and TWIST) in response to extracellular cues including cytokines and hypoxia.^1,2^ EMT-TFs activate multiple molecular pathways that ultimately leads to alteration in cytoskeleton and cell-adhesion proteins.^3^ EMT is a key early event in NSCLC biology and steers epithelial-like cells towards stemness, chemoresistance and metastatic dissemination.^4,5^ It is engineered through coordination of divergent molecular pathways,^6^ and presumably orchestrated by different EMT-TFs at different progression time points.^7–9^ How these pathways connect to each other and affect different modalities of EMT is still largely unexplored.

Thymidylate synthase (TS) is a de novo pyrimidine biosynthesis enzyme that catalyses the conversion of deoxyuridine monophosphate to thymidine monophosphate, essential for DNA synthesis and cell proliferation. It is targeted by chemotherapeutic drugs, like pemetrexed, in NSCLC, and has been widely studied as a chemoresistance marker.^10^ Our lab recently showed a correlation between TS expression and EMT markers in NCI-60 panel of cancer cell lines originating from different tissues^11^ and established its role in maintaining the de-differentiated mesenchymal-like state of triple-negative breast cancer.^12^ In this study we present evidence that TS is not a mere proliferation marker in NSCLC, but also has a direct role in driving EMT phenotypes, with several biological and clinical implications.

## Methods

### Cell lines

A549 (NCI), SK-MES-1 and Calu-1 (both ATCC) were cultured in RMPI-1640, supplemented with 10% FBS, 1%Pen/Strep and 1%l-Glutamine (all from Sigma). NIC-H23 cells were cultured in RMPI-1640, supplemented with 10% FBS, 1%Pen/Strep, 1%l-Glutamine and 1 mM sodium pyruvate (Sigma). LL/2, Ladi 3.1 and Ladi 2.1 cells were cultured in DMEM (Sigma) supplemented with 10% FBS, 1%Pen/Strep and 1%l-Glutamine. Human cells were STR-profiled, used between passages 3 and 15, examined for mycoplasma and maintained in Plasmocin (Invivogen) to prevent contamination.

### Lentiviral transduction

Plasmids for TS knock down (TRCN0000456666 for human cell lines and TRCN0000317583 for murine cell lines) are from Sigma. Scrambled pLKO.1 (referred to as pLKO) was used as control. Plasmids from *TYMS*-promoter reporter (HPRM33357-LvPM02), GAPDH promoter reporter (HPRM39787-LvPM02), TS expression vector (Ex-T0406-LV105b) and control vector (Ex-Neg-LV105b) are from GeneCopoeia. For production of lentiviral particles, 293T cells were transfected with 8 µg knock-down/expression vectors and 2 µg of pMDL, pVsVg and pRevRes in complex with 24 µg PEI (Polysciences). After 48 h, supernatant was collected, centrifuged and filtered. For transduction, 10^5^ cells were seeded in a six-well plate and infected in presence of 8 μg/ml polybrene (Sigma). Selection was done with 3 μg/ml puromycin (Sigma) and cells were maintained in 1 µg/ml puromycin.

### RNA sequencing

Total RNA was extracted using miRNeasy kit (Qiagen) following the manufacturer’s instructions. RNA-Seq libraries were constructed using the TruSeq sample Prep Kit V2 (Illumina). Briefly, 1 μg of purified RNA was poly-A selected and fragmented with fragmentation enzyme. After first and second strand synthesis from a template of poly-A selected/fragmented RNA, other procedures from end-repair to PCR amplification were done according to library construction steps. Libraries were purified and validated for appropriate size on a 2100 Bioanalyzer High Sensitivity DNA chip (Agilent Technologies). The DNA library was quantified using Qubit and normalised to 4 nM before pooling. Libraries were pooled in an equimolar fashion and diluted to 10 pM. Library pools were clustered and run on Nextseq500 platform with paired-end reads of 75 bases, according to the manufacturer’s recommended protocol (Illumina). Raw reads passing the Illumina RTA quality filter were pre-processed using FASTQC for sequencing base quality control. Sequence reads were mapped to UCSC human genome build using TopHat and differential gene expression determined using Cufflinks 2.1.1 and Cuffdiff2.1.1 as implemented in BaseSpace. The sequencing data has been submitted GEO dataset and could be accessed with GSE148589 accession number.

### Quantitative real-time PCR

Total RNA was extracted using miRNeasy kit (Qiagen) and 50 ng was converted to cDNA using Tetro cDNA synthesis kit (Bioline) with random hexamers. GAPDH was used as an internal control. TaqMan probes (Thermo-Fisher) were used for quantification in Applied Biosystems 7300. Fold change was calculated using the ΔΔCt method.

### Gene set enrichment analysis

Gene set enrichment analysis (GSEA), for computing overlap, on the differentially expressed genes upon TS knockdown was performed with the gene set collections in the Molecular Signatures Database v6.1 software. For EMT gene set enrichment analysis in the patient data, normalised gene expression values were downloaded from GEO database (GSE101929) and cbioportal platform for TCGA profile (LUAD, PanCaner). For calculation of TS Knockdown (KD) score, first, *z* scores of the down- and up-regulated genes upon TS knockdown were calculated. Then, the sum of z scores of downregulated genes was subtracted from the sum of z scores of upregulated genes and KD scores were obtained for each patient. Patients were grouped for the analysis based on either the median value of TYMS gene expression or KD score.

### Survival analysis

Normalised gene expression profiles of lung cancer samples were downloaded from GEO (GSE50081, GSE72094, GSE30219) and mRNA expression values as Z-scores were obtained for TCGA profiles (LUAD and LUSC) from cbioportal platform. Thirty-five samples from completely resected NSCLC patients were collected from the files of San Luigi Hospital, Orbassano, Turin, Italy. None of the patients received either neo-adjuvant chemotherapy or radiation therapy and all received adjuvant cisplatin and pemetrexed. All cases were reviewed and classified using anonymised samples. Clinical samples were stratified as TYMS-low and TYMS-high based on the median value of gene expression as cut-off. Kaplan–Meier estimate was used to generate survival curves and significance between the two groups were analysed using log-rank test in R software. Survival graphs from the KM Plotter database was generated based on TYMS expression by using the auto select best cut-off option. TS KD score for survival curve was calculated as described in the previous section.

### Western blot analysis

Cells were lysed in RIPA buffer and quantified using Pierce BCA kit (Thermo-Fisher). Proteins lysates (10–20 μg) were resolved on 10% SDS–PAGE gels and transferred to PVDF membrane (Thermo-Fisher). Membranes were blocked in 5% Milk (BioRad) in 1XTBS-T and incubated overnight in primary antibodies diluted in 5% milk at 4 °C. anti-TS (EPR4545) and -SPARC (SP205) antibodies were purchased from Abcam; anti-E- Cadherin (4A2), -Vimentin (D21H3), -AXL (C89E7), -FOSL1 (D80B4) and -β-Actin (8H10D10) were purchased from Cell Signaling. After incubation with secondary antibodies (Southern Biotech), the detection was performed using ECL (Thermo-Fisher) and developed on X-Ray film (Thermo-Fisher) using a chemiluminescence imager, AGFA CP100.

### Proliferation assay

For proliferation assay cells were seeded in 96-well plates in low density (5–20% initial confluency). Plates were loaded in IncuCyte-Zoom (Essen Bioscience) and scanned every 2–4 h. For each scan, phase contrast image was acquired from every well and was analysed by IncuCyte Zoom software.

### In vitro drug treatment

Pemetrexed was purchased from Sigma. For in vitro treatment cells were plated in a 96-well plate (4000 cells/well) and incubated overnight. For cytotoxicity death assay, 2000X Cytotox Green Reagent (Essen Bioscience) was diluted in RPMI and working dilutions of pemetrexed was prepared in Cytotox Green supplemented media. After treatment, plate was loaded in Incycuyte Zoom and images were acquired in real-time for phase to quantify growth. Activity of Cytotox reagent was simultaneously acquired at the green channel to quantify death. Incycuyte Zoom software was used for the analysis and data export.

### Migration assay

For migration assay cells were plated in 96-well plates so that they reach 90% confluency overnight. Cells were wounded using WoundMaker (Essen Biosciences) as per the instruction from the manufacturer. Plates were loaded in IncuCyte Zoom and were automatically scanned for programmed time interval. For each scan, wound width was recorded by the software and the proliferation inside the wound was normalised to the proliferation outside the wound, giving relative wound density for each time point.

### Tumoursphere culture

In all, 40,000 cells were seeded in triplicates in ultra-low attachment six-well plates (Corning) in complete Mammocult medium (Stem Cell Technologies), prepared according to the manufacturer’s instruction. After formation, spheres were counted by spinning at 300 g for 5 min and suspending in PBS (Lonza).

### siRNA transfection

Reverse transfection was done with Lipofectamine RNAiMAX Transfection Reagent (Thermo). 50 nM siRNA were mixed with 1.5 µl transfection reagent in 200 µl Opti-MEM (Thermo) and incubated for 15 min. After incubation transfection complex was added to the surface of a 12-well plates and 10^5^ cells, suspended in 800 µl, were added. Cells were incubated at 37 °C, 5%CO_2_. Cells were lysed for western blot after 72 h.

### In vivo experiments

C57BL/6 strain were used as experimental model to study effect of Ts-depletion in syngeneic LL/2 cells to prevent immune rejection.^13^ Mice were anaesthetised using isoflurane and euthanised by cervical dislocation.

For subcutaneous injections, 1 × 10^6^ cells resuspended in 50 µl 0.9% NaCl were mixed with Matrigel (Corning) in a ratio 1:1 (v:v). Cells were injected in right flanks of 10–15-weeks-old female C57BL/6, with eight mice per group. Calliper measurements were taken every 4th day and tumour volume was calculated using the formula (Length × Width^2^ × π)/6.

For tail-vein metastasis assay, 5 × 10^5^ LL/2 pLKO and shTs cells were resuspended in 100 µl PBS and injected in the tail vein of female C57BL/6, with 10 mice per group. Lung metastases were monitored by bioluminescence imaging (BLI) 4 weeks after injection. Anesthetised mice were intraperitoneally injected with 150 mg/kg D-luciferin (Kayman Chemicals). Bioluminescence images were acquired with Lumina III in vivo Imaging System (IVIS, Perkin Elmer). For all the mice exposure time was maintained at 180 s. Raw IVIS images were analysed with Living Image software and the metastasis was represented as radiance.

### Statistical analysis

Statistical tests were performed with the GraphPad software v.7 comparing groups of different conditions with replicates. In all tests, the statistical significance was set at *p* ≤ 0.05.

## Results

### TS is an essential NSCLC gene with prognostic/predictive power and correlates with EMT signatures

**Fig. 1:**
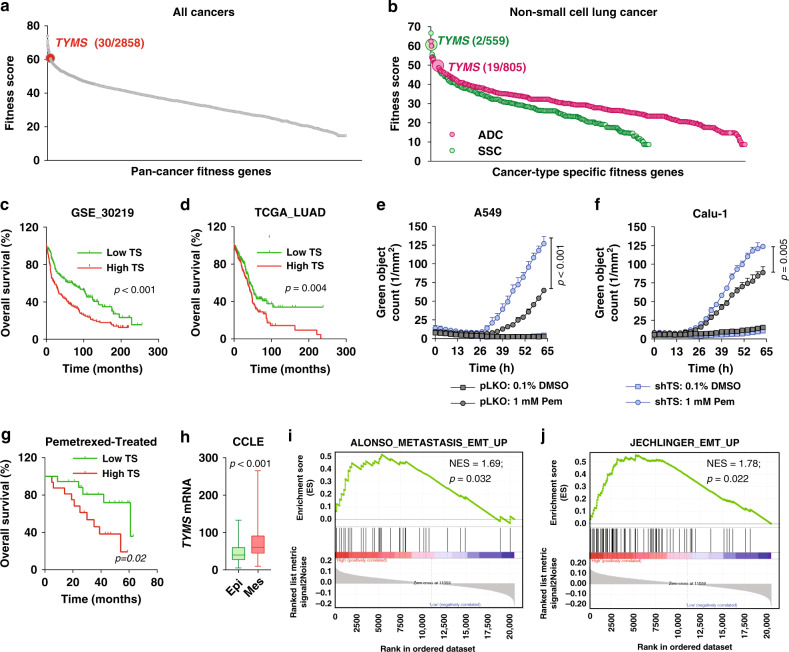
TS is an essential NSCLC gene with prognostic/predictive power and correlates with EMT signatures. **a** Graph showing TS ranking as a pan-cancer fitness gene for growth and survival. **b** Fitness score for TS in lung adenocarcinoma (ADC) and squamous cell carcinoma (SCC). Each dot represents a single gene. **c**, **d** Predicted overall survival in non-small cell lung cancer (NSCLC) patients separated according to the expression level of TS. *p*-value is Logrank test. Sensitivity of NSCLC cells lines A549 (**e**) and Calu-1 (**f**) to anti-folate drug pemetrexed (Pem) after shRNA-mediated knock-down of TS. Dead cells have been quantified as green object counts. *p*-value is represented as two-way ANOVA, Sidak’s multiple test. **g** Kaplan–Meier survival analysis of NSCLC patients treated with pemetrexed-based chemotherapy categorised according to high and low TS mRNA expression. *p*-value is Logrank test. **h** *TYMS* mRNA levels compared between NSCLC epithelial (Epi) and mesenchymal (Mes) cells from Broad Institute Cancer Cell Line Encyclopedia (CCLE) data, defined by *VIM*/*CDH1* mRNA expression ratio. *p*-value is from a Student’s *t*-Test. **i**, **j** Gene-set enrichment analysis of high and low TS correlating with published EMT gene signature (indicated on the top of the graphs) in lung adenocarcinoma subset of the Cancer Genome Atlas (TCGA) dataset. Error bars represent standard deviation.

We evaluated different clinical aspects of TS in NSCLC and assayed its correlation with EMT. As a rate-limiting de novo pyrimidine biosynthesis enzyme, *TYMS* (gene coding TS) has been proposed as an essential gene, but so far, no functional data have been shown in NSCLC. To evaluate dependency of NSCLC on TS, a dataset generated from a genome-wide CRISPR/Cas9 screen of 18,009 genes in 324 cancer cell lines was exploited.^14^ Based on a gene fitness score that defined how strongly a cancer is dependent on a gene for survival and growth, a priority score was generated to identify the most promising drug targets. Among all the pan-cancer fitness genes identified, TS ranked 30th (top 1%, Fig. 1a, see Supplementary Table 1 for the top 50 genes). In NSCLC subsets, it ranked 2nd and 19th in squamous cell carcinoma (SCC) and adenocarcinoma (ADC), respectively (Fig. 1b), indicating that NSCLC strongly depend on TS for sustained growth. TS was also found to be 5th among the pan-cancer priority targets identified (Supplementary Fig. 1A). TS was the only target of pemetrexed that exclusively appeared as significant in all the three lists (Supplementary Fig. 1A–C**)**. In concordance, TS expression has been consistently found increased in NSCLC compared to adjacent normal tissues^15^ and correlated with poor prognosis in different expression datasets analysed (Fig. 1c, d). TS is targeted by the anti-folate drug pemetrexed, and its overexpression has been proposed to determine chemoresistance.^16^ For in vitro validation, we established shRNA-mediated TS knockdown in two NSCLC cell lines and observed a significant increase in pemetrexed sensitivity (Fig. 1e, f). To test if in vitro evidence were also reflected in the outcome of chemotherapy-treated patients, we retrospectively analysed a small case-series of NSCLC patients treated with pemetrexed-based chemotherapy and found that higher TS gene expression significantly associated with worse prognosis (Fig. 1g). These results emphasise the importance of TS as a prognostic and predictive marker, in line with previous literature.^10^ However, chemoresistance is also an important hallmark of EMT, and recent pivotal findings from our lab associated TS expression with EMT markers in cancers from different origins and suggested a potential direct role.^11^ To test this in NSCLC, we analysed cells belonging to the CCLE dataset and found that lung cancer cell lines with high TS expression have enrichment in EMT signature genes (Supplementary Fig. 1F, G). When further categorised as epithelial or mesenchymal based on ratio of Vimentin (*VIM*) and E-Cadherin (*CDH1*) expression,^17^ (Supplementary Fig. 1H) mesenchymal-like cells expressed higher TS compared to epithelial-like (Fig. 1h). To further demonstrate its clinical significance, we investigated multiple datasets and found that patients with higher TS expression were significantly enriched for hallmark EMT genes (Fig. 1i, j and Supplementary Fig. 1I, J). These results indicate that TS is not only an essential proliferation gene with a strong prognostic and predictive role, but also has a potential power in EMT in NSCLC.

### Endogenous TS level is an important determinant of EMT phenotype

**Fig. 2:**
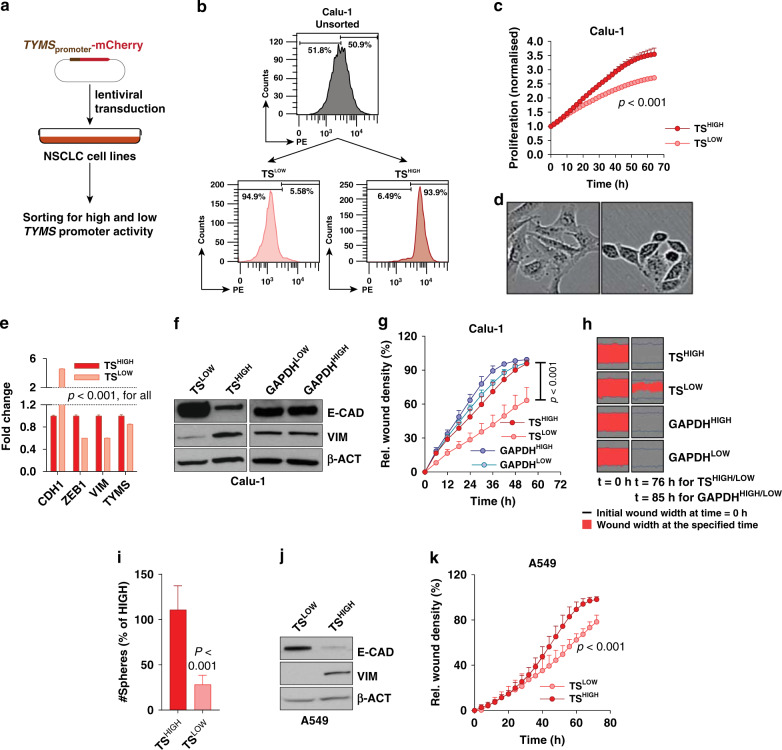
NSCLC cells can be sorted into distinct EMT phenotypes based on *TYMS* promoter activity. **a** Calu-1 cells were stably transduced with a lentiviral vector expressing mCherry gene under control of *TYMS* promoter. Transduced cells were selected for puromycin positivity and 5% Calu-1 cells were sorted with highest and lowest red fluorescence intensity. **b** FACS plots depicting the separation of high and low population of Calu-1 cells from scheme presented in **a**. The numbers on the FACS plots represent the percentage of TS^HIGH^ and TS^LOW^ population in the unsorted cells. **c** Real-time proliferation curves showing difference in growth between Calu-1 TS^HIGH^ and TS^LOW^ cells. Cells have been normalised to confluency at time 0, to exclude the difference in surface areas. *p*-value is represented as two-way ANOVA, Sidak’s multiple test. **d** Picture showing morphology of Calu-1 cells sorted for high *TYMS* promoter activity (TS^HIGH^) and low promoter activity (TS^LOW^). **e** mRNA quantification of *TYMS, CDH1, ZEB1* and *VIM* in Calu-1 TS^HIGH^ and TS^LOW^ cells. *p*-value is represented by multiple *t*-Test. **f** Protein quantification of E-CAD (marker of epithelial phenotype) and VIM (marker of mesenchymal phenotype) in TS^HIGH^ and TS^LOW^ Calu-1 cells. Cells were sorted in GAPDH^HIGH^ and GAPDH^LOW^ based on the scheme depicted in **a** but with mCherry transcript driven by GAPDH promoter. **g** Real-time proliferation-independent migration of cells from **f** in wounds created by IncuCyte wound-maker. *p*-value is represented as two-way ANOVA, Sidak’s multiple test. **h** Representative images from **g**. **i** Quantification of spheres formed by Calu-1 TS^HIGH^ and TS^LOW^ cells in low adherent cultures at low-seeding density. *p*-value is Student’s *t*-Test. **j** Quantification of E-CAD and VIM in A549 cells 5 days after sorting in TS^HIGH^ and TS^LOW^ based on the scheme in **a**. **k** Proliferation-independent real-time migration of A549 cells from **j** in a monolayer wound. *p*-value is represented as two-way ANOVA, Sidak’s multiple test. Error bars represent standard deviation.

TS expression has been shown to be highly varied in clinical samples stained for immunohistochemistry^15,18^ and bioinformatic analysis revealed that, within a tumour, individual cells can have extremely diversified TS expression (Supplementary Fig. 2A, B). When mapped to a published gene signature, individual cells with higher TS expression within a tumour showed a significant enrichment of EMT (Supplementary Fig. 2C). Hence, we postulated that intrinsic level of TS could be a strong determinant of EMT. To functionally validate the hypothesis, Calu-1 (a SCC cell line) was stably transduced with a promoter reporter construct^19^ that expressed mCherry fluorescent protein transcribed from *TYMS* promoter (Fig. 2a). After puromycin selection, cells were FACS-sorted for highest and lowest red fluorescence (indicated further as TS^HIGH^ and TS^LOW^, Fig. 2b). TS^LOW^ cells proliferated slower (Fig. 2c) and showed a distinct epithelial phenotype, whereas TS^HIGH^ resembled a mesenchymal-like morphology (Fig. 2d). To confirm differential EMT status at molecular level, expression of E-CAD and VIM, markers for epithelial-like and mesenchymal-like cells respectively, was quantified. At mRNA level, even with a minimal difference in TS expression, there was a striking difference between the expression of *CDH1* (gene coding E-CAD) and *VIM* (Fig. 2e). TS^LOW^ cells also expressed more E-CAD and lesser VIM compared to TS^HIGH^ cells at protein level (Fig. 2f), backed up by E-CAD changes observed in Calu-1 cells with knockdown and overexpression of TS (Supplementary Fig. 2D). Also, with the knockdown of TS, there was proliferation loss in the cells (Supplementary Fig. 2E) as observed with the cells after sorting. When assayed in proliferation-normalised wound migration assay, TS^LOW^ cells migrated slower than TS^HIGH^ cells (Fig. 2g, h). As a control for the promoter reporter assay, Calu-1 cells were sorted for GAPDH promoter activity and no difference in EMT markers and migration was observed in GAPDH^HIGH^ and GAPDH^LOW^ cells (Fig. 2f–h). TS^LOW^ cells also had reduced self-renewal capacity, quantified as the number of tumourspheres formed in a low-adherence culture (Fig. 2i). Further validation came from A549, that we had previously characterised to have lost stem cell phenotype after TS knockdown.^11^ When sorted in TS^HIGH^ and TS^LOW^ cells (Supplementary Fig. 2F), A549 cells, that exist in partial EMT state,^20^ recapitulated the EMT phenotypes observed in Calu-1 (Fig. 2j, k), although change in the self-renewal capacity was not observed (Supplementary Fig. 2G). We further independently validated TS-mediated EMT by knocking down TS in NSCLC cell lines SK-MES-1 (SCC cell line) and NCI-H23 (ADC cell line), where TS depletion led to upregulation of E-CAD and downregulation of VIM in SK-MES-1 (Supplementary Fig. 2H) and downregulation of VIM and ZEB1 (mesenchymal marker) in NCI-H23 (Supplementary Fig. 2I).

Interestingly, a rapid reversion of EMT phenotype was observed in the sorted Calu-1 cells, concomitant with the normalisation of *TYMS* promoter activity (Supplementary Fig. 2J). This was more evident in functionally distinct A549 cells, where the sorted cells showed higher TS levels in TS^HIGH^ a day after sorting, followed by a complete normalisation of TS and EMT markers after few passages (Supplementary Fig. 2K). Therefore, the phenotypic alterations observed between sorted cells were transient and in match with the differences in TS levels. These data strongly indicate a direct control of TS on EMT phenotype and hints that TS might have role to play in epithelial plasticity.

### TS regulates EMT genes in NSCLC

**Fig. 3:**
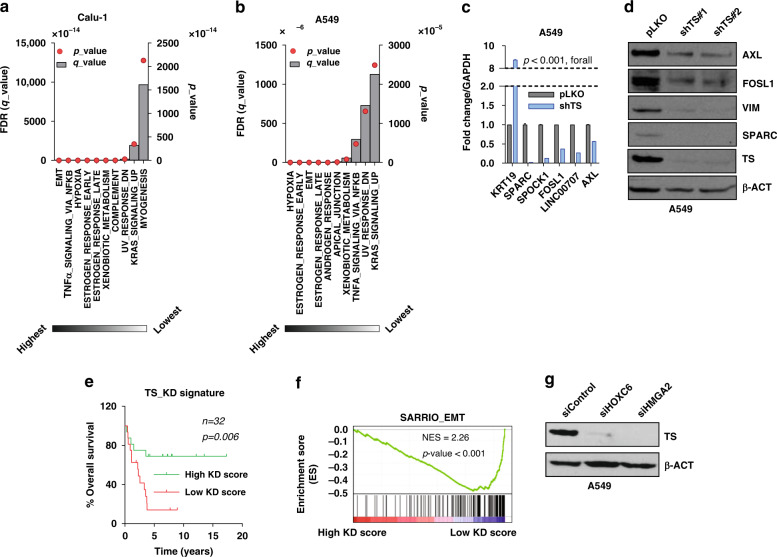
TS expression correlates with EMT gene signature. Gene set enrichment analysis of RNA-seq data of **a** Calu-1 cell sorted for high and low *TYMS* promoter activity and **b** A549 cells after transduction of shTS and scrambled pLKO backbone as control. **c** q-RT-PCR quantification of gene signature from A549 cells. *p*-value represents multiple *t*-Test. **d** Validation of TS-mediated downregulation of indicated genes at protein levels in A549 cells with TS knock-down. **e** Survival analysis of NSCLC patients from data set GSE101929 based on a knock-down score generated from A549 gene signature in **b**. *p*-value is represented as Log-rank (Mantel-Cox) test. **f** Gene set enrichment analysis with overlapping of TS knock-down score with EMT-signature in SCC patients from data set GSE4573. **g** A549 cells transiently transfected with 50 nM siRNA targeting transcription factors HOXC6 and HMGA2 identified from **a**.

Further, to identify the mediators of TS-promoted EMT, RNA was sequenced from Calu-1 TS^HIGH^ and TS^LOW^ cells in parallel with A549 cells with TS knockdown. Where Calu-1 sorted cells have shown growth reduction (Fig. 2c), A549 cells with TS knock-down proliferated normally (Supplementary Fig. 3A). Hence, two different cell lines that either have or don’t have a loss in proliferation, and that are processed by two independent techniques, were subjected to investigation. Pathway analyses consistently indicated EMT among the topmost differentially regulated pathways (Fig. 3a, b), confirming the EMT switch observed with E-CAD and VIM (Fig. 2f, j). KRT19, SPARC, SPOCK, LINC00707 (lung cancer promoting lincRNA), FOSL1 and AXL (identified as downstream targets of TS as they appeared in both signatures) were qPCR validated in both cell lines (Fig. 3c and Supplementary Fig. 3B). Of these genes, SPARC, FOSL1 and AXL, that have an established role in EMT in NSCLC,^21–23^ were strongly down-regulated at protein level in A549 cells with TS knockdown (Fig. 3d). Differentially expressed genes (DEGs) were used to derive a knockdown score, which predicted a worse survival associated with lower TS knockdown (higher TS levels, Fig. 3e) and correlated with published EMT gene signature (Fig. 3f). This indicated that TS-mediated EMT is empowered with its own prognostic impact, i.e. contributes to the adverse prognosis of NSCLC with high TS levels (Fig. 1c, d), suggesting a role for TS beyond proliferation. Several EMT transcription factors (HMGA2, HOXC6, SNAI2, SOX9, ARNTL2, SHOX6) were identified from DEGs in endogenously TS^HIGH^ and TS^LOW^ cells, from which siRNA mediated knock-down of HOXC6 and HMGA2 reduced expression of TS in Calu-1 (Supplementary Fig. 3C) and was validated in A549 (Fig. 3g). Thus, these results identified a network of TS mediated EMT, where HOXC6 and HMGA2 are upstream of TS and AXL, SPARC and FOSL1 are downstream mediators.

### Depletion of TS mitigates metastasis in vivo

**Fig. 4:**
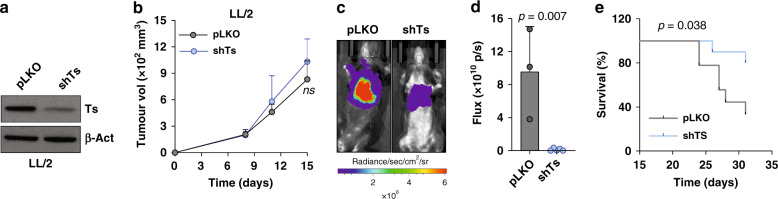
TS expression determines metastatic colonisation in vivo. **a** Western blot quantification of Ts in murine LL/2 cells. Scrambled pLKO has been used as a non-targeting control. **b** Quantification of primary tumour growth after subcutaneous injection of 1 × 10^6^ LL/2 cells from **a** in C57BL/6 mice (*n* = 8). *p*-value is represented as Student’s *t*-Test. **c**, **d** Quantification of metastatic localisation of luciferase expressing Ts knock-down and pLKO LL/2 cells in lungs of C57BL/6 mice. 5 × 10^5^ cells were injected in the tail-vein of mice (*n* = 10) and the luciferase activity was quantified by IVIS as readout for lung metastasis after 4 weeks. **e** Survival curves from mice in **d**. *p*-value is represented as Log-rank (Mantel-Cox) test. Error bars represent standard deviation.

Finally, in vivo approaches were used to confirm the role of TS on EMT and metastasis. Ts (mouse TS) expression was quantified in morphologically and functionally distinct mesenchymal-like (Ladi 3.1) and epithelial-like (Ladi 2.1) cells (Supplementary Fig. 4A, B), isolated from the same mouse model of NSCLC (p53^fl/fl-LSL^ KRAS^G12D/+^). Ts positively correlated with Vim and negatively with E-Cad (Supplementary Fig. 4C). Furthermore, to functionally evaluate in vivo effects of TS alteration on metastatic colonisation, *Tyms* gene was knocked down in murine Lewis lung carcinoma cell line LL/2 using stably transduced shRNA. A moderate Ts depletion (Fig. 4a) did not affect proliferation (Supplementary Fig. 4D), as we had previously determined from breast cancer cell lines, that TS needs to reduce beyond a threshold to diminish proliferation.^12^ Furthermore, Ts knockdown also did not hamper the growth of primary tumours from the cells subcutaneously injected in flanks of syngeneic mice (Fig. 4b). However, when injected in the tail vein, knocked down cells showed a highly significant reduction in lung metastatic colonisation (Fig. 4c, d), and the mice carrying cells with Ts depletion showed a significantly prolonged survival (Fig. 4e).

## Discussion

TS has been widely used as a chemotherapeutic target^24^ ascribed to its role in proliferation. This study experimentally validated this concept, as TS was one of the highest-ranked target gene identified based on the CRISPR/Cas9 screen analysed in this study. However, chemotherapeutic drugs that target TS might also mitigate other detrimental features associated with cancer. We confirmed the multifaceted role of TS in proliferation and chemoresistance and found a strong correlation with EMT gene signatures and prognosis, highlighting clinical, as well as biological relevance of TS in NSCLC. Interestingly, presented results clearly corroborate that different functions of TS can operate independently, as observed that TS depletion can mitigate EMT phenotypes without triggering proliferation loss. This we observed in two independent setups—first in A549, showing no loss in proliferation but alteration in EMT signature pathway after TS knockdown, and second in LL/2 cells that reduced metastatic colonisation after TS knockdown that didn’t affect proliferation and growth of primary tumour. It strongly adds to the proliferation-independent loss of differentiation that we had previously propounded,^12,25^ providing a strong rationale to revisit clinical and therapeutic aspects of TS in tumour biology and explore its therapeutic potential beyond proliferation.

Pemetrexed is used as a first-line treatment for NSCLC patients^26,27^ albeit response to the drug is remarkably varied. TS expression is an important determinant of sensitivity to pemetrexed^16,28^ and marks the worse clinical outcomes of pemetrexed treatment in NSCLC patients.^29^ In agreement to this, SCC generally respond poorly to pemetrexed as compared to ADC,^30^ partly attributed to higher TS expression in SCC compared to other histological subtypes.^15,31^ However, there is also a considerable variability to pemetrexed response within a given histological subtype.^32,33^ EMT could be an additional source of variability, as it is a key driver of chemoresistance against pemetrexed in NSCLC.^34^ In this study we have linked higher TS expression not only to pemetrexed outcome but also to EMT, indicating that TS has an important role in establishing a connection between EMT and pemetrexed resistance. This connection could also be extrapolated to genes that constitute the TS regulatory network, such as FOSL1, which regulates pemetrexed resistance in coordination with EMT-TF ZEB1.^22^ Therefore, a more inclusive biomarker signature needs to incorporate EMT genes and downstream genes like FOSL1, in addition to TS, for robust prediction of response to pemetrexed in NSCLC.^35^

TS expression has been previously shown to be stimulated by chemotherapy, as a cellular defence mechanism,^36^ and these data add the notion that chemotherapy-induced TS could lead to the adjustment of EMT phenotypes in patients, that, in turn, might influence the efficacy of treatment. This aspect could be taken into consideration for the implementation of therapeutic strategies combining EMT-suppressing drugs and chemotherapy, or for the future design of the next generation of TS-inhibitors, which should not enhance TS levels.

In NSCLC, EMT enhances the inflammatory tumour microenvironment leading to activation of multiple immune checkpoint proteins, including PD-L1.^37^ A recent clinical trial has demonstrated a better outcome in NSCLC when pemetrexed is administered in combination with a PD-L1 inhibitor, pembrolizumab.^38^ Since, TS drives EMT and EMT has been shown to modulate response to immune therapy, a functional correlation between TS expression and susceptibility to immunotherapy could be deduced in NSCLC. In fact, we identified interleukins such IL-6, IL-7 and IL-32 in our TS signatures, which have been previously linked with poor prognosis and metastasis in NSCLC.^39–41^ Hence, a follow-up study is needed to validate this correlation, as the two drugs are frequently combined.

The present study also underscores the plasticity of cancer cells with mixed EMT population, as was reported in cells sorted for high and low TS expression (Supplementary Fig. 2J, K). It could be interesting to understand how TS mechanistically drives plasticity by molecular profiling of cells at several time points between the sorting and phenotype reversal.

We furthermore establish the role of TS in metastasis, where TS knock-down abrogated the metastatic colonisation and improves mice survival without affecting proliferation and growth of primary tumour. This observation indicates that TS strongly influences the success against selection pressure at the metastatic site. Further retrospective validation in patients can establish TS as a metastasis marker in NSCLC.

Finally, this study provides a perspective for a network that could integrate different signalling pathways to effectuate various aspects of cancer progression that are mediated by TS (Supplementary Fig. 4E), worth further investigation. Different transcriptional regulators and effector proteins identified in this study have an established role in EMT in NSCLC and connect with master EMT-TFs. HMGA2, for instance, affects proliferation and metastasis by regulating TWIST,^42^ FOSL1 regulates chemotherapy, exogenous SPARC promotes invasion and metastasis by activating SNAI1^21,42^ and AXL activates TWIST to affect cell cycle.^23^ A follow-up study could further substantiate TS as an integration point for these pathways resulting in a cumulative readout in terms of metastasis.

Thus, this study provides strong evidence that TS, apart from proliferation enzyme, also regulates EMT in NSCLC. Targeting EMT-related processes could represent a promising therapeutic strategy to suppress the aggressiveness of TS-overexpressing NSCLC.

## Supplementary information

Supplementary Material

## Data Availability

Data from RNA-seq has been submitted to GEO database (Accession Number: GSE148589)
