# Supplementary material for: Thymidylate synthase drives the phenotypes of epithelial-to-mesenchymal transition in non-small cell lung cancer
Source: Br J Cancer. 2020 Oct 7;124(1):281–9. doi: 10.1038/s41416-020-01095-x (PMC7782507; doi:10.1038/s41416-020-01095-x)
Supplement: Supplementary file 1 — Supplementary Material [file 41416_2020_1095_MOESM1_ESM.docx]

**
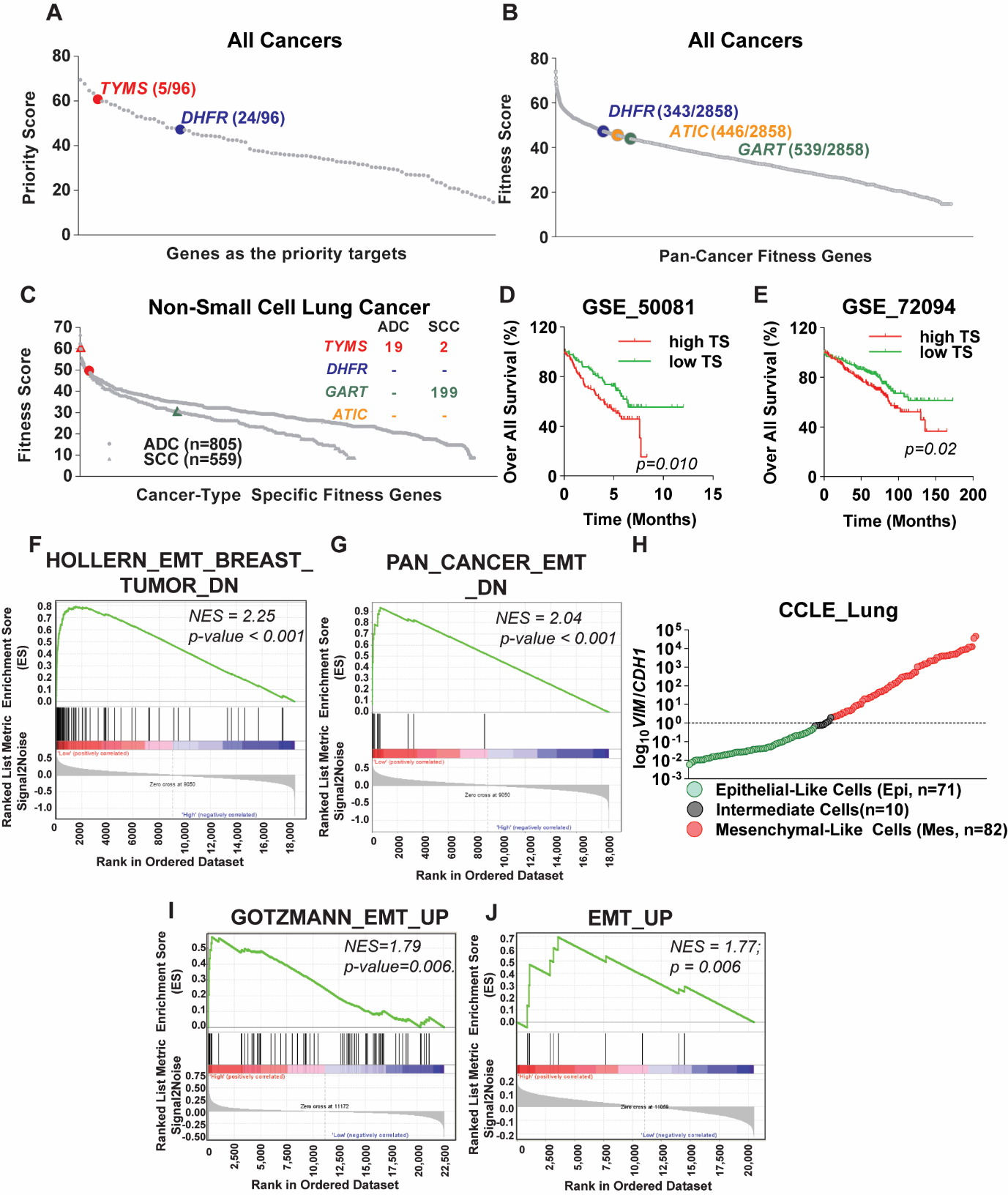
**

**Supplementary figure 1**

**
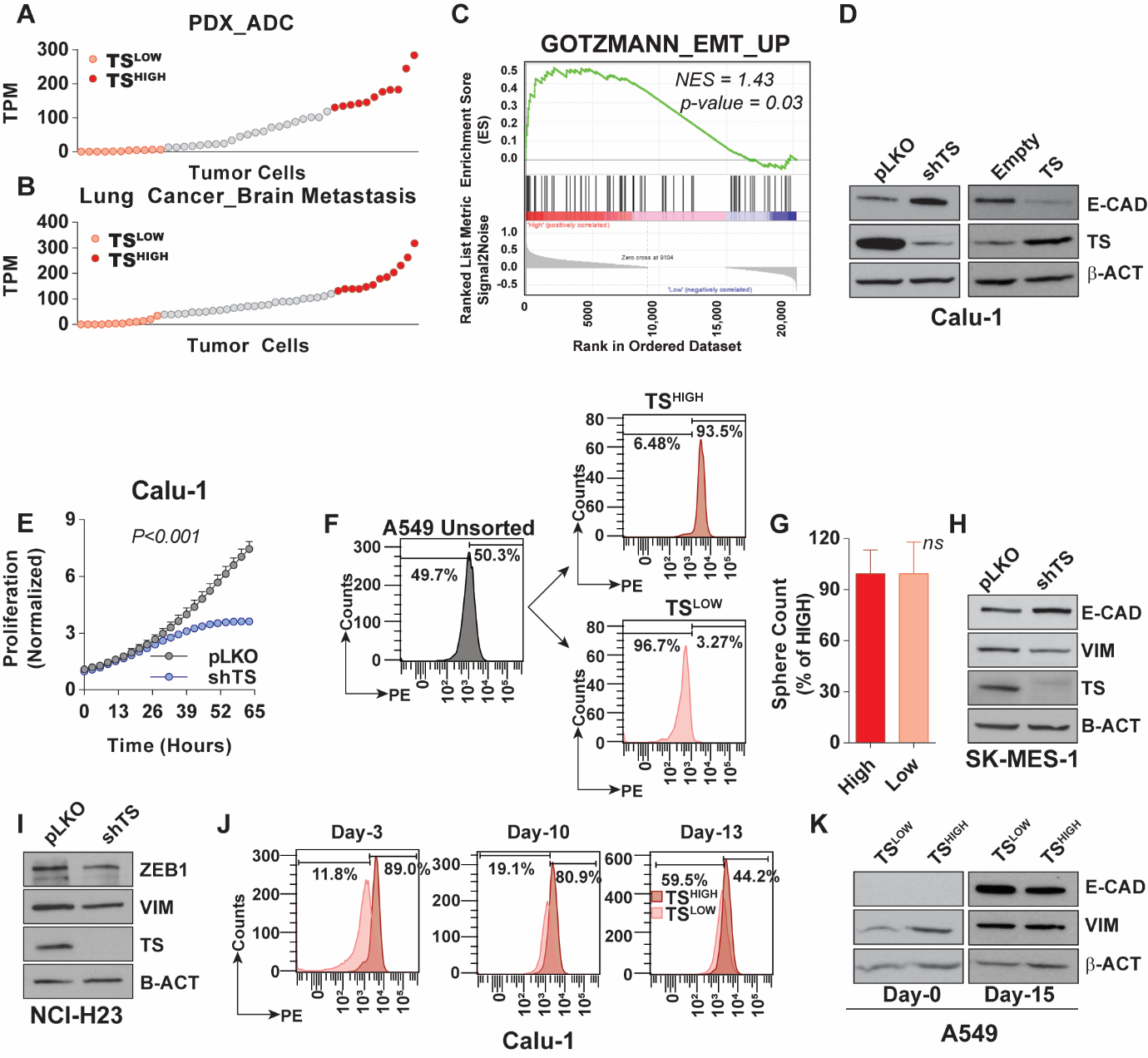
**

**Supplementary figure 2**

**
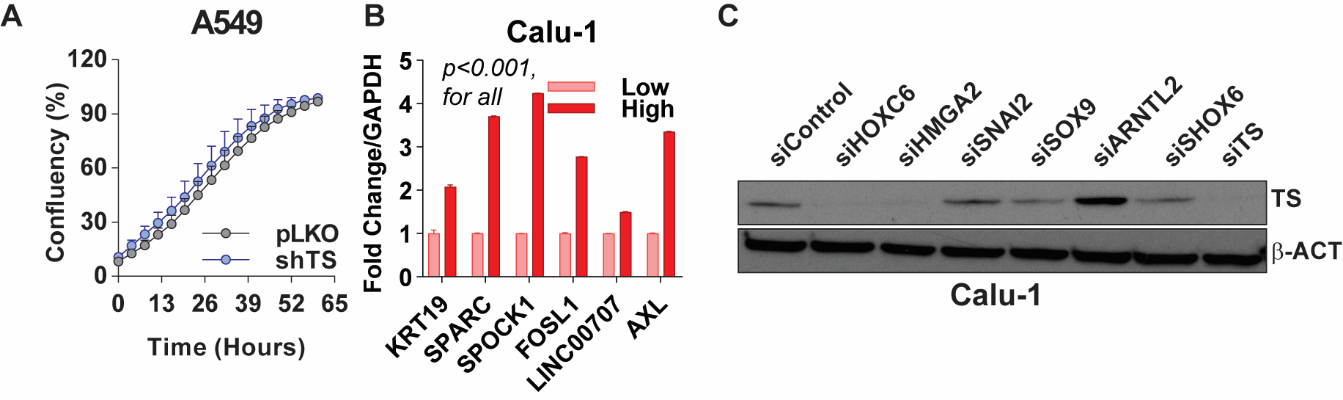
**

**Supplementary figure 3**

**
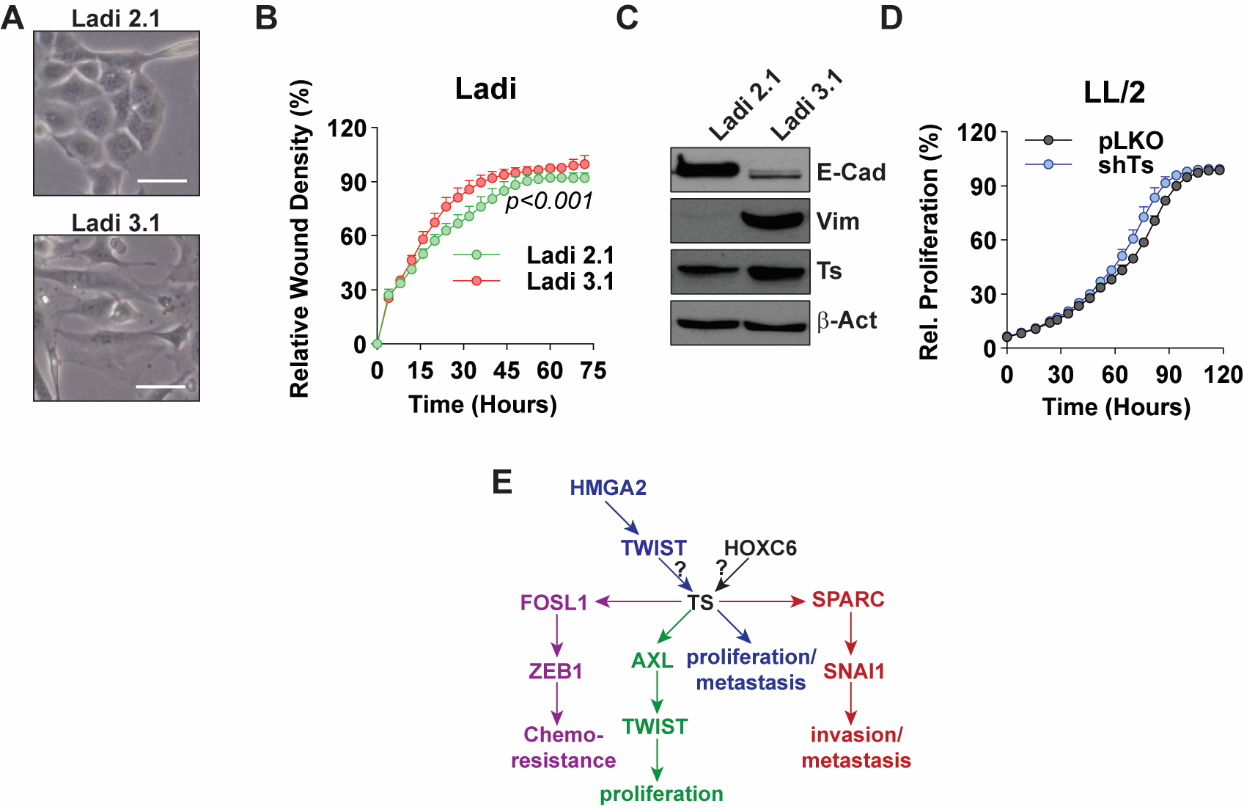
**

**Supplementary figure 4**

**Supplementary figure Legends**

**Supplementary Figure 1. TS is an essential NSCLC gene with prognostic/predictive power and correlates with EMT signature.** (**A**) Scores for *TYMS* and other pemetrexed targets in list of pan-cancer priority targets. (**B**) Graph showing fitness rank of pemetrexed targets in all cancer types and (**C**) non-small cell lung cancer (NSCLC) subset*.* Scores for individual target has been tabulated in the table for NSCLC. A gene absent from the list of the fitness gene is denoted by ‘-’. *DHFR* (dihydrofolate reductase); *GART* (phosphoribosylglycinamide formyltransferase); *ATIC* (5-aminoimidazole-4-carboxamide ribonucleotide formyltransferase/IMP cyclohydrolase). (**D-E**) Predicted overall survival NSCLC patients separated according to the expression level of TS in mentioned datasets. P-value is Log-rank test. (**F-G**) Gene-set enrichment analysis of high TS expressing NSCLC cell lines from CCLE for enrichment of EMT signature. (**H**) NSCLC cell lines arranged in epithelial-like, intermediate and mesenchymal-like phenotype bases on the ratio of *VIM/CDH1*. (**I-J**) Gene-set enrichment analysis of high and low TS samples in GEO dataset GSE72094 and in the (Cancer Genome Atlas TCGA) lung adenocarcinoma expression dataset, respectively. Names of the mapped EMT gene signatures have been indicated on the top of graph.

**Supplementary Figure 2. Endogenous expression of TS is determinant of EMT phenotype.** Graph representing expression of *TYMS* mRNA from individual cells from (**A**) patient derived ADC and (**B**) brain metastasis of lung cancer from single-cell RNA sequencing (scR-Seq). Each dot represents a single cell. Cells in the lowest and highest quartile have been classified as TS^LOW^ and TS^HIGH^ respectively. (**C**) GSEA of scR-Seq data showing significant correlation between individual cells from a tumour expressing high TS and EMT. (**D**) Quantification of TS and E-CAD in Calu-1 cells with shRNA mediated knock-down and overexpression of TS. Scrambled pLKO and empty vector have been used as controls. (**E**) Real time proliferation assay comparing proliferation between pLKO and Calu-1 with TS knockdown. p-value is represented as 2-way ANOVA, Sidak’s multiple test. (**F**) FACS plots depicting the separation of high and low population of A549 cells sorter for *TYMS* promoter activity. The numbers on the FACS plot represent the percentage of TS^HIGH^ and TS^LOW^ population in the unsorted cells. (**G**) Quantification of spheres in A549 cells sorted in TS^HIGH^ and TS^LOW^. P-value represents student’s t-Test. (**H**) Western blot from SK-MES-1 cells depicting the changes in the expression of E-CAD and VIM. (**I**) Quantification of VIM and ZEB1 (markers of mesenchymal phenotype) in NCI-H23 cells after TS knockdown. No E-CAD was detected in these cells. (**J**) FACS quantification of Calu-1 cells showing normalization of TS promoter activity. (**K**) Western blot of lysates from indicated time points showing reversal of EMT phenotype in A549 TS^HIGH^ and TS^LOW^ cells. E-CAD is a trypsin sensitive membrane cell-adherence protein and could not be detected in sorted cells in suspension on day 0. Error bars represent standard deviation.

**Supplementary Figure 3. Upstream and downstream genes were identified in NSCLC.** (**A**) Real-time quantification of proliferation in A549 cells after TS knockdown. (**B**) mRNA quantification of genes identified from RNA-seq overlapping in Calu-1 TS^HIGH^ and TS^LOW^ cells and A549 shTS cells. p-value represents multiple t-Test. (**C**) Western blot screening of transcription factors expressed differentially in Calu TS^HIGH^ and TS^LOW^ cells (Figure 3A). Calu-1 cells were transfected with 50µM siRNAs. siRNAs that reduced the expression of TS at protein levels were identified as its potential upstream regulator. Error bars represent standard deviation.

**Supplementary Figure 4. TS expression determines Metastasis in vivo.** (**A**) Pictures showing morphological distinction in Ladi cells isolated from a NSCLC mouse model. Ladi 2.1 is a cell line with epithelial trait whereas Ladi 3.1 cells harbour mesenchymal features. (**B**) Real-time quantification of migration difference between Ladi 2.1 and Ladi 3.1 cells (**C**) Western blot showing difference in Ts and EMT markers in Ladi cells. (**D**) Graph comparing proliferation rates between LL/2 shTs and pLKO cells. p-value is represented as 2-way ANOVA, Sidak’s multiple test. Error bars represent standard deviation. (**E**) A perspective hypothesis depicting potential upstream regulators and effectors of TS mediated EMT and their possible integration. HMGA2 and HOXC6 upregulate the expression of TS, where TS modulates the expression of proteins (AXL, FOSL1 and SPARC) that have been shown to have a role in EMT. Pathways and the responses elicited by each protein have been depicted in a different colour.

**Supplementary Table 1: List of fitness score for top 50 genes**

| **Priority Score** | **Gene** | **description** | **family** | **pubmed_id** |
| --- | --- | --- | --- | --- |
| 74.108 | SEC61A1 | Sec61 translocon alpha 1 subunit | | 10212142\|18480044 |
| 73.750 | CREBBP | CREB binding protein | Zinc fingers ZZ-type\|Lysine acetyltransferases | 8413673 |
| 71.296 | KRAS | KRAS proto-oncogene, GTPase | RAS type GTPase family |  |
| 69.528 | HDAC1 | histone deacetylase 1 | Histone deacetylases, class I\|EMSY complex\|NuRD complex | 8602529 |
| 68.226 | HMGCS1 | 3-hydroxy-3-methylglutaryl-CoA synthase 1 | |  |
| 68.053 | MAT2A | methionine adenosyltransferase 2A | | 1426236\|9703951 |
| 67.692 | MET | MET proto-oncogene, receptor tyrosine kinase | Receptor Tyrosine Kinases | 1846706\|1611909\|25941349 |
| 67.230 | ANAPC10 | anaphase promoting complex subunit 10 | Anaphase promoting complex | 10318877\|11230166 |
| 67.192 | CCND1 | cyclin D1 | Cyclins | 1826542\|1833066 |
| 66.125 | IRF4 | interferon regulatory factor 4 | | 8921401\|18417578 |
| 65.742 | TPX2 | TPX2, microtubule nucleation factor | | 9207457\|10393424 |
| 65.594 | CCND2 | cyclin D2 | Cyclins | 1386335 |
| 65.417 | NUP85 | nucleoporin 85 | Nucleoporins | 8124707 |
| 65.058 | MYCN | v-myc avian myelocytomatosis viral oncogene neuroblastoma derived homolog | Basic helix-loop-helix proteins |  |
| 64.688 | ERBB2 | erb-b2 receptor tyrosine kinase 2 | CD molecules\|Minor histocompatibility antigens\|Erb-b2 receptor tyrosine kinases |  |
| 64.332 | PPP2CA | protein phosphatase 2 catalytic subunit alpha | Protein phosphatase catalytic subunits | 8383590 |
| 63.968 | PFDN5 | prefoldin subunit 5 | Prefoldin subunits | 9630229\|9792694 |
| 63.938 | SOX10 | SRY-box 10 | SRY-boxes | 9462749\|10441344\|12944398 |
| 63.773 | WRN | Werner syndrome RecQ like helicase | Exonucleases\|RecQ like helicases | 9288107 |
| 63.605 | EGFR | epidermal growth factor receptor | Erb-b2 receptor tyrosine kinases | 1505215 |
| 63.341 | EIF4A1 | eukaryotic translation initiation factor 4A1 | DEAD-box helicases | 8493113\|9790779 |
| 63.055 | ALDOA | aldolase, fructose-bisphosphate A | | 3570299 |
| 62.633 | TBCD | tubulin folding cofactor D | |  |
| 62.464 | UBE2C | ubiquitin conjugating enzyme E2 C | Ubiquitin conjugating enzymes E2 | 9122200 |
| 62.459 | LONP1 | lon peptidase 1, mitochondrial | AAA ATPases\|Proteases, serine | 8248235\|8119403 |
| 62.159 | RAE1 | ribonucleic acid export 1 | WD repeat domain containing\|Nucleoporins | 9370289\|9256445 |
| 61.832 | SEH1L | SEH1 like nucleoporin | WD repeat domain containing\|Nucleoporins | 12196509\|14517296\|15146057 |
| 61.366 | NUDC | nuclear distribution C, dynein complex regulator | NudC family |  |
| 61.147 | SNRNP25 | small nuclear ribonucleoprotein U11/U12 subunit 25 | | 15146077 |
| 60.867 | TYMS | thymidylate synthetase | |  |
| 60.552 | TUBGCP6 | tubulin gamma complex associated protein 6 | | 11694571\|11258795 |
| 60.000 | ATP5A1 | ATP synthase, H+ transporting, mitochondrial F1 complex, alpha subunit 1, cardiac muscle | F-type ATPases\|Mitochondrial complex V: ATP synthase subunits | 1830491 |
| 59.888 | TUBB4B | tubulin beta 4B class IVb | Tubulins | 3999141 |
| 59.776 | PIK3CA | phosphatidylinositol-4,5-bisphosphate 3-kinase catalytic subunit alpha | Phosphatidylinositol 3-kinase subunits | 1322797 |
| 59.770 | EIF4G1 | eukaryotic translation initiation factor 4 gamma 1 | Parkinson disease associated genes | 1429670\|9372926\|21907011 |
| 59.596 | DNM2 | dynamin 2 | Pleckstrin homology domain containing | 7590285\|9143510 |
| 59.469 | CTNNB1 | catenin beta 1 | Armadillo repeat containing | 7829088 |
| 59.329 | TBCB | tubulin folding cofactor B | | 8978778 |
| 59.000 | CDH1 | cadherin 1 | CD molecules\|Type I classical cadherins | 9925936 |
| 58.833 | KLF4 | Kruppel like factor 4 | Zinc fingers C2H2-type\|Kruppel like factors | 9422764\|16372018 |
| 58.783 | TRIM37 | tripartite motif containing 37 | Ring finger proteins\|Tripartite motif containing | 9106536\|10888877 |
| 58.632 | MCL1 | BCL2 family apoptosis regulator | BCL2 family | 7682708\|7835896 |
| 58.500 | CEBPA | CCAAT/enhancer binding protein alpha | Basic leucine zipper proteins\|CCAAT/enhancer binding proteins | 1535333\|1840554 |
| 58.396 | EXOSC9 | exosome component 9 | Exosome complex |  |
| 58.347 | CCNE1 | cyclin E1 | Cyclins | 1833066 |
| 58.327 | STAG1 | stromal antigen 1 | Cohesin complex | 9305759 |
| 58.250 | ESR1 | estrogen receptor 1 | Nuclear hormone receptors | 3754034 |
| 58.228 | RPP25L | ribonuclease P/MRP subunit p25 like | | 16998185 |
| 58.000 | POU2AF1 | POU class 2 associating factor 1 | | 8617501 |
| 57.926 | CDC37 | cell division cycle 37 | | 8703009\|8666233 |

**Supplementary Table2: List of differentially regulated genes in Calu1 TS^HIGH^ and TS^LOW^ cells**

**Genes Upregulated**

| SCARB1 | HS6ST1 | THSD4 | IDH1 | LINGO1 |
| --- | --- | --- | --- | --- |
| RAB27B | AC016205.1 | AC090204.1 | CUX1 | DUSP16 |
| MFF | IDH3A | AC135048.1 | ITPRIP | LINC00963 |
| CLIP2 | PIM1 | CYB5D2 | EPHB4 | SPP1 |
| FSBP | NUDT7 | ZNF653 | GMDS-AS1 | CRAT |
| TRAF4 | GSTP1 | SHARPIN | SERPINB1 | XPR1 |
| TP53TG1 | TRUB2 | KIF13B | GJA1 | GSR |
| CARMIL1 | MAPRE2 | SNHG22 | ENO3 | BAG1 |
| LHX4 | THRB | CEBPB | LPCAT1 | WWP1 |
| ALDH3B1 | TST | TRIML2 | P4HA3 | PTGES2 |
| PDXK | MTFMT | OLFML2A | PARD3 | ENDOG |
| UBAC1 | GPR157 | ZNF532 | BIRC7 | ZNRF3 |
| PPT2 | RAP1GAP2 | RASSF2 | NDUFA8 | HMG20B |
| RPL30 | METTL7B | PON2 | ZFP36L2 | COL4A6 |
| CD24P4 | ASNS | DNMT3B | SEMA3B | ZNF618 |
| OR7E14P | FTCDNL1 | C17orf51 | PITX1 | CRMP1 |
| SGK2 | CHEK2 | JAG1 | C16orf74 | AC091390.4 |
| NINJ1 | LINC01503 | KANK1 | EPB41L4B | SH3BP5 |
| KLHDC10 | HSPB8 | CTSL | DGAT1 | GGT1 |
| EEF1A2 | NEDD9 | SVEP1 | TRIB3 | CHAC1 |
| ZHX1 | PXN-AS1 | IER5L | PCDH9 | ABCA7 |
| FZD7 | MDH2 | RHBDD2 | HSPB1 | NOTCH1 |
| GNE | LRSAM1 | PDCD4 | PC | LIFR |
| SORT1 | MISP | C1S | EFNA1 | TPPP3 |
| ADAM15 | SUCLG2 | RETREG1 | AC026979.3 | CTSD |
| ACAT2 | ESRRA | PARVB | JAKMIP3 | GPX4 |
| E2F2 | FNBP1 | CHP1 | LY6E | LINC02057 |
| LAMA5 | MTMR11 | EFNA4 | C9orf3 | HSD3B7 |
| APTR | PHF7 | TMEM168 | GTF2IRD1 | DPP7 |
| CNTNAP3B | PPP3CC | AZIN1-AS1 | RHOB | TBC1D31 |
| ZNF275 | UAP1L1 | ANKS6 | C1R | VWA1 |
| NAA38 | CASP10 | CELSR2 | PLCH1 | ABLIM1 |
| SNN | LTBP1 | INTS10 | TWIST2 | DGCR5 |
| SLC27A4 | NDUFB9 | MROH6 | SAPCD2 | FAM83H |
| PAQR6 | IMPA2 | SPSB2 | DENND3 | ARRDC1 |
| HK1 | ALAS1 | LRIG3 | COL4A5 | EGFL7 |
| GSN | INO80C | AC007240.1 | AMDHD1 | RAB26 |
| LGR4 | DHRS13 | PGD | CDKL5 | CA12 |
| IRS2 | GPRC5A | DECR1 | AC009779.2 | LMO7 |
| SGCE | RN7SK | ESCO2 | PKP3 | SLC29A4 |
| TMEM189 | GADD45B | FBXO44 | SLC16A5 | BMPER |
| TMEM205 | P2RX5 | CBLB | GINS4 | FZD5 |
| HS1BP3 | DAPK1 | RELL2 | NECAB3 | TPD52 |
| ARSE | HIP1R | SLC25A4 | RAB15 | SPAG1 |
| UBALD2 | CYB5A | PIR | S100A4 | CCNYL1 |
| TP53I11 | NNMT | PFKFB2 | FDX1 | EPAS1 |
| CXCL16 | LAPTM4B | MSI2 | NR0B1 | SLFN11 |
| FAM47E-STBD1 | AS3MT | DHCR7 | ZP3 | AC009118.2 |
|  |  |  |  |  |

| DERL1 | B4GALT4 | SUCLG2-AS1 | CLU | DKK1 |
| --- | --- | --- | --- | --- |
| SLCO4A1 | OSGIN1 | GLP2R | GLDC | CFH |
| RGS20 | PIK3CD-AS2 | COMTD1 | LINC01322 | CPVL |
| ACOX2 | FCHO1 | LINC01433 | RNF19A | HAS2 |
| CYC1 | TM4SF1 | SLC29A2 | NDRG2 | HHIPL2 |
| GSTM4 | IGFBP1 | NT5M | MGLL | OAS1 |
| SFI1 | RAB31 | MEGF9 | KLF4 | GAL3ST1 |
| AVPI1 | KIFC2 | AP1S3 | KRT8P3 | CTH |
| PBK | THEM6 | KRT86 | NPR3 | SYT1 |
| AGPAT2 | DSCC1 | CFD | KRT85 | SLC45A4 |
| IFITM3 | ABCB6 | MYRF | FIBCD1 | PALMD |
| AK1 | NQO2 | FA2H | UNC13D | MYO15B |
| ACSS2 | LLGL2 | TFPI2 | IL1R1 | ALDH1L2 |
| CHCHD10 | GOT1 | DIRAS3 | RAB20 | NR4A1 |
| TESC | MFAP3L | LINC01137 | IGFBP2 | AKR1B15 |
| DLK2 | GNG11 | KIAA0319 | TENM3 | BCHE |
| SLC23A2 | SLC22A18 | MFSD12 | DMTN | AKR1C1 |
| TOB1 | IGFBP3 | TMEM184A | LGSN | MAP7 |
| BMP8B | FOXC1 | ADAMTS9 | PPFIBP2 | NID2 |
| CAVIN2 | TBC1D2 | C2orf82 | ERRFI1 | MTUS1 |
| NBEAL2 | SLC6A9 | STOM | C1QTNF6 | INSL4 |
| SCD | SEC14L4 | CDA | CSGALNACT1 | WISP2 |
| SLC8B1 | MAFK | CYP2S1 | OPLAH | CA2 |
| SMOC1 | PDE3A | RHOBTB1 | FAM167A | MUC13 |
| ATP1B1 | ABCA2 | RAET1G | KAZALD1 | C11orf86 |
| GRAMD1B | MREG | ARRB1 | KRT87P | VCAN |
| ZNF714 | RAB3D | PLA2G4A | SYT12 | SAMD11 |
| NEDD4L | PTGR1 | ASB4 | DHRS2 | CLDN2 |
| LINC01703 | PDE8B | SCARA3 | SELENBP1 | CEACAM6 |
| PON3 | AC008738.1 | NUP50-AS1 | SH3TC1 | AKR1B10 |
| BCO1 | FAM19A5 | RHCG | SLC7A2 | FGG |
| LACTB2 | PMP22 | AC008443.5 | HMOX1 | VTN |
| RHOBTB2 | GATS | CYP1B1 | GDF15 | NOTCH3 |
| GPRIN3 | RHOU | FLVCR2 | ALDH3A1 | FST |
| ARHGAP27 | CCDC159 | AL162411.1 | RAP1GAP | FGL1 |
| C9orf172 | ELF3 | TSPAN13 | PEG10 | PTPRD |
| AL355075.4 | CRB3 | JUP | CD55 | UGT1A9 |
| SRP14-AS1 | GLRX | KRT8 | FSTL4 | MUC5B |
| DUSP5 | LRRN2 | RPS29P16 | POF1B | CPLX2 |
| CD38 | SPTBN2 | AL139819.1 | AKR1C2 | PPARGC1A |
| CNNM2 | CACNA2D1 | NRCAM | DOK4 | AC105999.2 |
| L1CAM | AC006077.2 | ADSSL1 | NME1-NME2 | DEFB1 |
| PLXND1 | PCK2 | EPB41L1 | RNF43 | UGT1A6 |
| KCNK1 | CCND3 | IL20RB | CDH1 | AGR2 |
| ZNF385A | FAM69B | KRT81 | ANG | GPX2 |
| AC068580.4 | SGK1 | KRT18 | CYP26B1 | S100P |
| POR | SREBF1 | IGFL2-AS1 | RHOV | PDK4 |
| PDLIM5 | IFI30 | ASS1 | AKR1C3 | FAM83A |
| FXYD2 | FGA | FGB | RTN4RL2 |  |
|  |  |  |  |  |

**Genes Downregulated**

| AL136295.6 | SNORA73B | PIK3CD | DNMBP |
| --- | --- | --- | --- |
| EVI2B | BCL2A1 | CHGB | AC139795.2 |
| AL512598.1 | FYN | STK26 | A1BG |
| RNU1-60P | CADPS2 | SHOX2 | LCOR |
| NPY4R2 | NT5E | LTBP2 | AC015982.1 |
| ZBED2 | MOB3B | FOXL2 | FOXD1 |
| PKIA | TCEAL3 | CTGF | ARNTL2 |
| AC025580.2 | TINAGL1 | MAN2B1 | PTX3 |
| AL365356.5 | ZNF544 | GSPT2 | CDIP1 |
| MECOM | WNT5A | AC073389.1 | SYNJ2 |
| SNAI2 | RTL8B | KDELC1 | FUT8 |
| AC012065.3 | SPOCK1 | NIPAL4 | EBI3 |
| AC006504.3 | IL11 | NFE2L3 | IRAK2 |
| AC010809.1 | COL5A1 | FHL2 | MT1A |
| IL7 | AL354714.2 | APOL1 | PADI2 |
| ADRB2 | ZNF239 | TMEM59L | ZNF827 |
| AFF3 | TNFRSF9 | STAC | GLIPR1 |
| AC013451.2 | GPR176 | PPP1R13L | C15orf52 |
| IGFBP7 | GTF2IP12 | CLGN | C14orf105 |
| CDH4 | LHFPL6 | LRRFIP1P1 | NOP14-AS1 |
| PDE2A | RUNX2 | XDH | AC010422.3 |
| FPR1 | BTNL9 | GBP1 | AC002310.1 |
| KCNQ3 | TNRC6C | TRAM1L1 | PCDHGC3 |
| ITGB3 | MATN3 | AXL | SP6 |
| PRR16 | SOX7 | LFNG | PROSER1 |
| ALOX5AP | ZNF283 | CDCP1 | PID1 |
| LOXL1-AS1 | LIMA1 | JPH3 | PNMA8A |
| OXCT1 | ADPRH | ANXA8L1 | LRP4 |
| LRMDA | NNT | TANC2 | NUDT11 |
| BACE1-AS | SPHK1 | TSPAN5 | AL121748.2 |
| ADGRL2 | CA8 | ZNF585B | INSIG1 |
| CORO2B | ITGBL1 | KIRREL3 | SOX9 |
| C5orf17 | SAMD9 | CCL2 | PMEPA1 |
| IGFN1 | GLUD2 | ETS1 | FAM66C |
| LINC01515 | ZNF22 | UGT8 | HOXC6 |
| NPTX1 | MAGEH1 | ZNF586 | HAPLN3 |
| ANPEP | MAGEA6 | GPAM | LSR |
| SEMA7A | COL4A1 | EPHB2 | CAMK2N2 |
| WISP1 | MYZAP | CXCL1 | TNC |
| COL13A1 | GSTM3 | ATP1A3 | ZMAT3 |
| RBP1 | SLC10A4 | CRTAC1 | DMKN |
| LINC02086 | PORCN | AK5 | DSE |
| PADI3 | FHOD3 | SOCS3 | MLF1 |
| MAN1A1 | ZNF548 | ZNF112 | PLEKHA2 |
| CNRIP1 | NUAK1 | ZNF215 | WDR70 |
| AC090409.1 | SLC12A8 | APBB2 | TRIO |
| OTUB2 | MT2A | G0S2 | COL6A1 |
| ADAMTS12 | CES3 | IFI27L2 | ELK3 |
| AOX1 | ZNF697 | ZMIZ1-AS1 | ADARB1 |
| AC008687.6 | A1BG-AS1 | ANKRD13A | CCND1 |
| HK2 | PDGFB | CLMP | C15orf48 |
| AC124283.3 | TTC3P1 | COL4A2 | EVC2 |
| CHRNA9 | COL6A2 | ZNF551 | BX322562.1 |
| RAB3B | RASSF10 | SRPX | RASA3 |
| CSMD3 | MOXD1 | MCOLN2 | ZNF32 |
| SPOCD1 | SH2B3 | TPM1 | LRRC8C |
| OSGEPL1-AS1 | SORCS2 | TMEM158 | RUNX1 |
| NKILA | GALNT9 | GXYLT2 | AL512488.1 |
| ADAM19 | GFRA1 | AC073896.2 | ATOX1 |
| AC004585.1 | LAMC2 | AC010326.4 | FHDC1 |
| MAP3K7CL | FRMD6 | ARAP2 | ACER2 |
| IL6 | CDK14 | KIF5C | ZNF606 |
| TCEAL8 | ARMCX3 | TIPARP | ITGAX |
| CSF2 | PODXL | LINC02274 | ITGA2 |
| AL096865.1 | AL138756.1 | ALOXE3 | CATSPER1 |
| AC243773.2 | CALB2 | RNF182 | FAM111A |
| AC015674.1 | VANGL2 | TRAF1 | MARCH4 |
| TG | ZNF569 | PRTFDC1 | ZNF776 |
| PLEK2 | GBP3 | TNFAIP6 | GOLGA7B |
| CRISPLD2 | KIAA1549L | ZNF222 | AC020915.2 |
| LAMA4 | PLCXD2 | FAM228B | AC027031.2 |
| AC008536.1 | CD82 | AL590617.2 | USP49 |
| KCTD12 | SLIT3 | ARMCX6 | STK17A |
| FBXO32 | FRMD4A | TSPYL5 | USP31 |
| TENM2 | ZNF185 | BDNF | ZNF8 |
| EFNB2 | MEG3 | CPQ | SUPT3H |
| WDR66 | SLFN5 | AC131206.1 | LINC01116 |
| ABLIM3 | NNT-AS1 | SMTN | GLIPR2 |
| CCBE1 | PCDHAC2 | CXCL8 | SAMD4A |
| ZNF155 | ANKRD44 | LINC00511 | MFGE8 |
| SDC2 | AFAP1L2 | LYPD1 | NDST1 |
| ACTL8 | SERPINE1 | ZNF565 | SPINT2 |
| FBLN1 | ZNF223 | CLTCL1 | DCUN1D3 |
| FLG-AS1 | LINC01006 | SMURF2 | COG3 |
| RRAD | HMGA2 | LRRC75A | SP100 |
| HBEGF | SFMBT2 | NAV3 | SLC16A2 |
| TLE1P1 | TNFAIP3 | TOB2P1 | INPP4B |
| CTHRC1 | MPP1 | HRH1 | ZNF773 |

| ZNF674-AS1 | AP003486.1 | AL050341.2 | GNA13 |
| --- | --- | --- | --- |
| DRAM1 | LTBP3 | QPCT | JRKL |
| RBMXL1 | SLC25A21-AS1 | PLAU | UXS1 |
| ADAM12 | ZNF561 | SNX19 | CASK |
| CTSS | F11R | LINC01588 | CASC15 |
| B4GALT6 | GTF2F2 | LINC00941 | C17orf100 |
| ZNF324B | ZNF529 | CAVIN1 | HENMT1 |
| GNB4 | C1GALT1C1L | DIAPH3 | DOCK1 |
| STRA6 | PRELID2 | ZSCAN30 | PLEKHG3 |
| DBP | CYR61 | IRAK1BP1 | MYO10 |
| AC135050.6 | ZNF567 | PDZK1IP1 | RAB11FIP2 |
| SHB | SLC6A17 | PGS1 | VPS36 |
| ITGA5 | ARHGEF7 | AP4S1 | PIGM |
| SPDL1 | TIMP2 | HIPK2 | KIRREL1 |
| SMIM8 | ZFP36L1 | TSPYL4 | WDFY2 |
| HERC5 | USP40 | MTND6P4 | FBN1 |
| CNOT6 | MNS1 | LINC00842 | NFKB1 |
| C3orf52 | EIF3J-AS1 | NOCT |  |
| IL32 | CYLD | SDC4 |  |
| RCBTB2 | RNF2 | ANXA6 |  |
| PDLIM7 | LINC00847 | PHLDA1 |  |
| GPALPP1 | OCLN | MOK |  |
| CDC14A | ANKRD10 | MAP3K4 |  |
| KLF7 | DTX3 | ELF4 |  |
| CD274 | CHAMP1 | ZFP90 | PPP4R1L |
| ITGA3 | SLC26A2 | ZNF146 | AC018629.1 |
| JUN | RAB8B | BCAR3 | ZNF710 |
| AC008870.2 | FAM111B | NXPE3 | KPNA3 |
| MT1X | PLEKHG4 | CENPBD1 | PLK2 |
| C16orf45 | LRRC6 | VPS37B | ENTPD7 |
| HDGFL3 | MCAM | ZNF460 | VMP1 |
| AC093616.1 | FNDC3A | CLEC2D | AC008741.2 |
| SOBP | TMEM238 | HEG1 | SH3KBP1 |
| RASD2 | RIN3 | GAL | ZMIZ1 |
| EHD3 | CHST7 | TRIM21 | SMAD3 |
| ZNF354B | TTC30B | PTPN1 | ALKBH8 |
| ZNF226 | ZNF30 | ARL4C | BTBD11 |
| TCF7L1 | C20orf194 | ZFAND4 | LINC00707 |
| CYTH1 | RCAN1 | DLEU1 | SLC25A37 |
| AC124798.1 | ESD | MYCBP2 | TMX3 |
| NFKBIZ | MCL1 | CRYBB2P1 | PIP4K2B |
| ZNF585A | REV3L | STK40 | IPO5 |
| BACH1 | ASB16-AS1 | RPGR |  |
| ZNF230 | PEAR1 | CD44 |  |
| CCDC186 | UVRAG | SLC6A15 |  |
| HOXB13 | FAT4 | TMCC2 |  |
| DNAJB5 | ZC3H13 | CASC3 |  |
| ZNF550 | ZNF45 | APLF |  |
| C6orf223 | ZNF503 | TBC1D4 |  |
| PDGFC | ZNF70 | TMEM246 |  |
| ZNF331 | PLEKHO2 | IRGQ |  |
| ERCC2 | MICAL1 | MARC1 |  |
| AC112220.4 | ZBTB18 | PFDN1 |  |
| TBC1D10A | G3BP1 | SGPL1 |  |
| LY6K | ZNF180 | GNPTAB |  |
| ALX1 | AC098614.1 | TMTC4 |  |
| ZNF300 | GNA12 | LPIN2 |  |
| ZEB1 | PAM | FAM3C2 |  |
| ADAM22 | MYH9 | RRAS2 |  |
| TNIP1 | LINC01232 | AATF |  |
| MACROD2 | FTSJ1 | SERTAD2 |  |
| MYBL1 | CCDC18 | GPX3 |  |
| SMS | MBNL2 | KDM4B |  |
| DOPEY2 | AMIGO2 | WBP4 |  |
| UCN2 | ZNF101 | CHFR |  |
| C3 | DNER | ARHGAP10 |  |
| AC006058.1 | AC022431.1 | SPRED2 |  |
| BIVM-ERCC5 | TMEM132A | SPRY2 |  |
| ALPK2 | AC025265.1 | SETDB2 |  |
| BIRC3 | AL136164.4 | KIF3C |  |
| TRIM34 | DZIP1 | NPIPP1 |  |
| PTPN14 | UCHL3 | IGF2BP2 |  |
| ACO1 | DDX10 | SAP30BP |  |
| SLC9A7 | GGACT | DIS3 |  |
| LRIG1 | LINC00973 | NARF |  |
| SPEG | OCIAD2 | DYRK3 |  |
| CCDC33 | BBX | AC139887.2 |  |
| ZNF260 | AC087481.3 | PXN |  |
| HAS3 | AHRR | C1QTNF1 |  |
| ALCAM | CACHD1 | INAFM2 |  |
| AC115837.1 | PEA15 | NRG1 |  |
| FOXO1 | MRAS | FGD6 |  |

**Supplementary Table3: List of differentially regulated genes in A549 shTS and pLKO cells**

**Genes Upregulated**

| AC078909.2 | C1orf61 | BCRP3 | EFCAB7 |
| --- | --- | --- | --- |
| RNU6-33P | CDH4 | AC068631.2 | LYPLAL1 |
| AC005498.2 | PHF21B | ZNF853 | ZNF607 |
| SPARC | GOLM1 | FGFBP1 | REXO5 |
| AC010997.5 | ADGRG1 | PIBF1 | CKS2 |
| AP001469.2 | AC097376.2 | FAM72C | AC243960.2 |
| HIST2H2AB | TSC22D1 | DPYSL3 | C9orf85 |
| LINC00479 | BCAR3 | CEND1 | TFAP4 |
| ACAP1 | NNMT | NPM1P24 | SERTAD1 |
| AC009469.1 | AL162411.1 | ZFP28 | CCDC150P1 |
| AC008878.1 | TRIML2 | FOSL1 | FLNB |
| ALOX5AP | ZRANB3 | KIRREL3 | TTF1 |
| PRDM16 | AC016705.2 | AC006058.3 | USP12 |
| AL355512.1 | SLC25A45 | SYT13 | KLF4 |
| AL645608.5 | GAR1 | AC018629.1 | CAMLG |
| SPOCK1 | LINP1 | COL5A1 | MYH9 |
| AL606834.3 | MSRA | FCGBP | NDUFAF2 |
| LINC01978 | TRPM2 | AC017033.1 | RABEP2 |
| LMO1 | SFN | NPY4R | FAM213B |
| CASC8 | AC010969.2 | HOXA7 | AXL |
| TSPAN1 | TIMP2 | CA8 | HID1 |
| AL365356.5 | TBXAS1 | TAF13 | WAC-AS1 |
| PRMT5-AS1 | TAGLN | TNS4 | CDK2AP1 |
| SIK1 | UCHL3 | AC004585.1 | AC092718.4 |
| SNRPGP15 | RRAS2 | AL583722.1 | ADGRE5 |
| CALB2 | CTNNAL1 | ZNF469 | AC005034.3 |
| PYCARD | PLEKHA2 | RASSF10 | C1orf21 |
| HMGA1P8 | INTS13 | AP000347.1 | MPHOSPH6 |
| AC003002.1 | PHF19 | DOK3 | MTSS1L |
| AL445222.1 | RWDD1 | PIN4P1 | AC007485.2 |
| LINC00707 | HPCAL1 | RPS24P8 | PRIMPOL |
| AC034102.1 | SUMO2 | AC004967.1 | AL391056.1 |
| MUC5AC | SNRPE | AL162386.2 | NOP14-AS1 |
| AC145207.5 | AL359183.1 | FAM84B | TYMS |
| LINC00941 | NDUFAF6 | SLC9A7 | LRRC8A |
| LYPD1 | RPL22L1 | LAMC2 | AL031775.1 |
| ARSI | ZNF324B | MT1X | AC012184.3 |
| PDZK1IP1 | RP11-343N15.5 | STMN3 | DUSP7 |
| KCNMA1 | CAVIN1 | EDN2 | SLC25A51 |
| POLN | YAE1D1 | CHRNA10 | ZNF205 |
| NEXN | OTUB2 | AC048341.3 | POLE3 |
| PREX1 | UBE2W | RPS3AP5 | COA6 |
| PHOSPHO2 | ERH | MARCH4 | PTGES3 |
| ZNF185 | EFEMP1 | VIM | PCED1B |
| JCAD | ZNF8 | CRIP2 |  |
|  |  |  |  |

**Genes Downregulated**

| TMEM144 | TCN2 | KIF12 | SCD | LRRC75B |
| --- | --- | --- | --- | --- |
| P4HA3 | BCL2L2-PABPN1 | AFF1 | AGMAT | GRB7 |
| CHEK2 | MEX3A | PKD1L2 | SLC9A3R1 | FGFR4 |
| TIRAP | PCSK9 | CTH | ARID3A | GALC |
| CXXC5 | ID1 | AC141586.1 | SUMF1 | NUDT11 |
| TUBAL3 | AP000769.1 | CDIP1 | TMEM254 | ZNF641 |
| TDRKH | FGB | ADGRG2 | VAMP1 | ID2 |
| ING3 | AC099336.2 | FGL1 | CRELD1 | SH3BP5 |
| C12orf73 | IQGAP2 | DNMT3B | GSAP | AKAP12 |
| HNRNPUL2-BSCL2 | C1QL4 | SAMD11 | NCR3LG1 | CAMK2N1 |
| ZNF76 | AKR1C2 | HILPDA | FAM174A | ZNF516 |
| GAPLINC | HSPA2 | YPEL3 | CTDSPL | SMPDL3B |
| CA11 | AP002360.1 | SGK1 | CYP27B1 | WDR91 |
| ZNF397 | SELENBP1 | EPS8 | PAM | ANG |
| ST3GAL2 | HCN3 | NPR3 | KIAA1161 | C1orf115 |
| IGF1R | ADM | DUSP16 | FBXO41 | MTRNR2L12 |
| TLN2 | HOXA-AS2 | GCHFR | CLK4 | TWIST2 |
| CRB3 | AMACR | HGD | PON3 | AL138963.3 |
| ACVR2B | RAP1GAP2 | TM4SF4 | SLC30A3 | SYBU |
| METTL12 | ID2-AS1 | SYNPO2 | IL32 | DOK4 |
| NUP50-AS1 | BTN3A2 | CDH1 | FRAS1 | ADCY9 |
| C17orf97 | RAB20 | CRYBG2 | JUP | CLCN5 |
| PAQR7 | GCA | RAB37 | DMXL2 | ZC3HAV1L |
| EPS8L1 | LINC00632 | CPS1 | DKK1 | DZIP1L |
| B4GALNT1 | METTL7B | VTN | HSD17B14 | SYTL4 |
| ZNF485 | CXCL2 | GAL3ST1 | PHLPP1 | SCARA5 |
| FAM234B | MR1 | ANXA13 | HSD3B7 | PTP4A3 |
| RNF123 | SARM1 | LARGE1 | KREMEN1 | ID4 |
| AC007240.1 | HOXA6 | HNF4A | COLCA2 | CCDC18-AS1 |
| EPDR1 | ANXA4 | NBR2 | MMP7 | BAAT |
| SLC12A6 | NDRG1 | ANKS4B | TOR1AIP1 | EXOC6 |
| KIAA1217 | CYP2R1 | SRR | IGFBP3 | ALX1 |
| ZFYVE1 | LIPH | CRYM | SLC51B | ARRB1 |
| CSAD | CLDND2 | PLIN2 | MICB | FAM13A |
| SNAI1 | MAGEA6 | CP | IRAK2 | STAT4 |
| BBS2 | GDAP1 | SLC16A4 | SH3RF2 | AL590617.2 |
| INSIG1 | FGFR1 | GABARAPL1 | R3HDM2 | AL021707.6 |
| SESN2 | ERRFI1 | ADAMTS9 | AC008429.1 | AL133351.4 |
| USP25 | TMEM175 | SERPINA6 | ZHX2 | C8orf4 |
| KLHL8 | TMEM37 | SLC7A7 | TBX2-AS1 | SRPX2 |
| DNAJB9 | FAM66C | FBXO27 | APLP1 | CACNA1G |
| ALPK1 | SDHAF4 | APOH | PTPRG | BMP4 |
| ADORA2B | SELENOM | CIDEC | LINC-PINT | KIF21B |
| C3orf52 | LINC00265 | DFNA5 | HLA-DMA | APOL2 |
| LGALSL | TPGS1 | CA9 | DAB2 | OLFML3 |
| PBX1 | SULT2B1 | PDK4 | BDKRB2 | SPX |
| EPS8L3 | CGN | FGFR3 | DEFB1 | USH1C |
| TENM3 | SLC2A3 | F5 | KRT19 | UPK1B |
| PDZK1 | TMCC1 | TRIM31 | LIN7A | SNHG25 |
| FBXO2 | VASH2 | SLFN11 | AC105243.1 | |
| FZD8 | VCAN | DCDC2 | TM4SF20 |  |
| FOS | PTGS2 | PRODH2 | NTS |  |
| CNKSR2 | AC092336.1 | HLA-DMB | NEFL |  |
